# Localized Contrast-Induced Encephalopathy of the Medulla Oblongata Following Endovascular Treatment of Posterior Circulation Lesions: A Report of Two Cases

**DOI:** 10.7759/cureus.77512

**Published:** 2025-01-15

**Authors:** Hiroki Kobayashi, Yuichi Nomura, Naoki Oka, Jouji Kokuzawa, Yasuhiko Kaku

**Affiliations:** 1 Department of Neurosurgery, Asahi University Hospital, Gifu, JPN

**Keywords:** coil embolization, contrast-induced encephalopathy, contrast materials, endovascular treatment, posterior circulation

## Abstract

Contrast-induced encephalopathy (CIE) is a rare but serious complication of endovascular treatments. Contrast materials can disrupt the blood-brain barrier and subsequently cause encephalopathy. We herein report two cases. Case 1 is a 63-year-old woman presented with an unruptured right vertebral artery aneurysm. The patient underwent the stent-assisted coil embolization of the lesion and experienced dizziness, nystagmus, and numbness in the right upper limb after the procedure. T2-weighted images and fluid-attenuated inversion recovery sequence (FLAIR) images revealed a high-intensity lesion in the right lateral medulla oblongata. The lesion was thought to have vasogenic edema and was diagnosed as CIE. Case 2 is a 76-year-old man presented with severe basilar artery stenosis. The patient underwent percutaneous transluminal angioplasty and stenting of the basilar artery and experienced dizziness and dysarthria during the procedure. Computed tomography (CT) revealed a high-density lesion in the left lateral medulla oblongata. According to the water-iodine image of dual-energy CT, the lesion was thought to be a contrast leakage and was diagnosed as CIE. In the two cases, intravenous steroids improved the symptoms, and follow-up imaging revealed the disappearance of the lesions. Frequent injections, large amounts, low temperatures, and stagnation of the contrast material in the same vascular territory can induce encephalopathy. Patients with chronic kidney disease, hypertension, and lesions of the posterior circulation can be at risk of CIE.

## Introduction

Contrast-induced encephalopathy (CIE) is a rare but serious complication of endovascular treatment [1-5]. CIE presents with neurological deficits and seizures following the administration of contrast agents [1-5]. Contrast materials can disrupt the blood-brain barrier (BBB) and subsequently cause encephalopathy [2-4]. Imaging such as CT and MRI are important to exclude thromboembolic and hemorrhagic complications and to confirm the diagnosis. Most symptoms related to CIE disappear within 48-72 hours, and most patients with CIE have a good prognosis. Several factors, such as the large amount of contrast material, can induce encephalopathy, but details are unclear [6,7]. There have been few reports of localized CIE of the medulla oblongata. We herein report two cases of localized CIE of the medulla oblongata following endovascular treatment for lesions of the posterior circulation.

## Case presentation

Case 1

**Figure 1:**
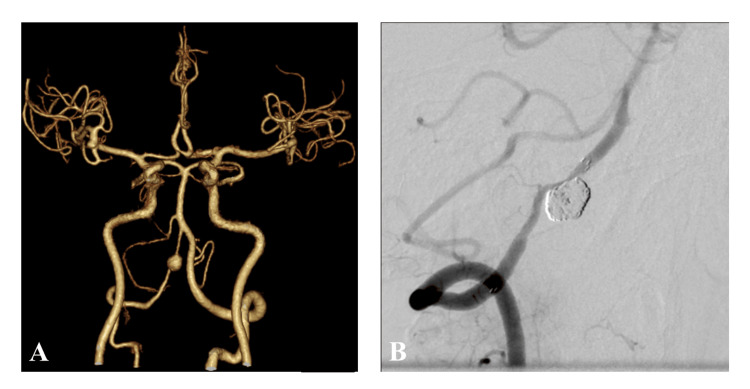
3D computed tomography angiogram (CTA) and digital subtraction angiography (DSA) of Case 1 A: 3D CTA shows an unruptured aneurysm (8 x 7 mm) of the right vertebral artery. B: DSA obtained just after the stent-assisted coil embolization of the aneurysm demonstrates a successful coil packing of the aneurysm and good patency of the right vertebral artery.

A 63-year-old woman had a history of a small right middle cerebral artery aneurysm, and annual follow-up magnetic resonance imaging (MRI) has been conducted since 2004 in Asahi University Hospital, Gifu, Japan. MRI revealed de-novo, an unruptured right vertebral artery aneurysm, in 2023. The patient's physical examination and laboratory data were unremarkable. The patient's medical history included hypertension. The patient had a normal renal function with 102 mL/min/1.73m^2^ of estimated glomerular filtration rate (eGFR). Magnetic resonance angiography (MRA) and three-dimensional (3D) computed tomography angiogram (3D CTA) revealed an unruptured aneurysm of the right vertebral artery (8 x 7mm) (Figure 1A). Stent-assisted coil embolization of the lesion was accomplished by introducing a 5-Fr guiding sheath into the right vertebral artery using a Neuroform Atlas stent 3 mm in diameter x 21 mm in length (Stryker Neurovascular, Fremont, CA, USA) and a total of 48 cm of platinum coils (target coil, Stryker Neurovascular, Fremont, CA, USA). The procedure was completed uneventfully with successful occlusion of the aneurysm (Figure 1B). The duration of the procedure was 58 minutes, and 80 ml of the contrast material was used. The contrast material was kept warm at 37°C during the procedure.

**Figure 2:**
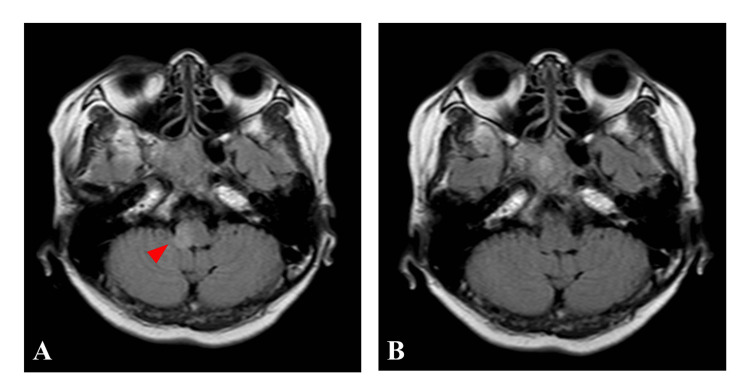
Post-operative images of Case 1 A: Fluid-attenuated inversion recovery sequence (FLAIR) images of MRI obtained one hour after the procedure show a high-intensity lesion in the right lateral medulla oblongata. B: A follow-up FLAIR image of MRI obtained 17 days after the procedure shows no high-intensity lesion in the right lateral medulla oblongata.

The patient experienced dizziness, nystagmus, and numbness in the right upper limb one hour after the procedure. Diffusion magnetic resonance imaging (MRI) demonstrated no ischemic lesions. T2-weighted images and fluid-attenuated inversion recovery sequence (FLAIR) images, however, revealed a high-intensity lesion in the right lateral medulla oblongata (Figure 2A). The lesion was thought to have vasogenic edema and was diagnosed as CIE. The patient received 1000 mg of methylprednisolone for two days. The symptoms improved within a week, and the lesion in the right lateral medulla oblongata disappeared on MRI 17 days after the procedure (Figure 2B).

Case 2

**Figure 3:**
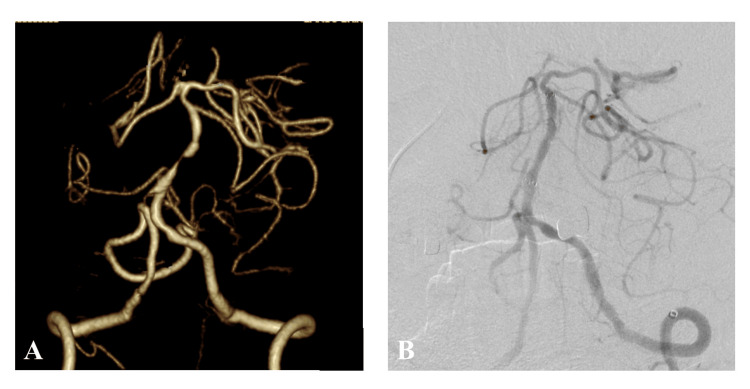
3D computed tomography angiogram (CTA) and digital subtraction angiography (DSA) of Case2 A: 3D CTA of Case 2 shows severe stenosis of the basilar artery. B: DSA obtained just after stenting of the basilar artery demonstrates a complete dilatation of the basilar artery.

A 76-year-old man was diagnosed with severe basilar artery stenosis on screening MRA before coronary artery bypass grafting and was referred to Asahi University Hospital in 2023. The patient's physical examination and laboratory data were unremarkable. The patient's medical history included hypertension, diabetes mellitus, and coronary heart disease. The patient had a normal renal function with 70.5 mL/min/1.73 m^2^ of eGFR. 3D CTA demonstrated a severe stenosis of the basilar artery distal to the antero-inferior cerebellar arteries (Figure 3A). Percutaneous transluminal angioplasty and stenting of the basilar artery were accomplished by introducing a 5-Fr guiding sheath into the left vertebral artery using a Wingspan stent (Stryker Neurovascular, Fremont, CA, USA) (Figure 3B). The duration of the procedure was 29 minutes, and 115 ml of the contrast material was used. The contrast material was kept warm at 37°C during the procedure. 

**Figure 4:**
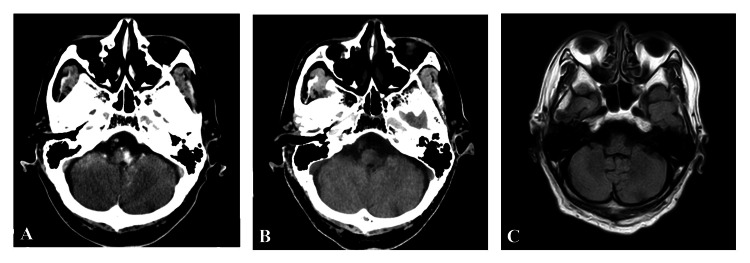
Post-operative images of Case 2 A: Plain CT immediately after the procedure shows a high-density lesion in the left lateral medulla oblongata. B: Water-iodine image of dual-energy CT shows no lesions. C: T2 image of MRI obtained one day after the procedure shows no lesions.

The patient experienced dizziness and dysarthria during the procedure. Computed tomography (CT) revealed a high-density lesion in the left lateral medulla oblongata (Figure 4A). The lesion was thought to be a contrast leakage according to the water-iodine image of dual-energy CT (Figure 4B) and was diagnosed as CIE. The patient received 1000 mg of methylprednisolone for two days. Symptoms improved within a few days, and follow-up CT and MRI demonstrated the disappearance of the lesion (Figure 4C). 

## Discussion

CIE is a rare but serious complication of endovascular treatments [1-5]. CIE was first reported in 1970 after the cardiac catheterization. The incidence of CIE ranges between 0.3% and 1.0% [7]. The precise mechanism and causes of CIE, however, remain unclear. Contrast materials can induce disruption of the BBB and subsequently cause encephalopathy, leading to neurological deficits due to the neural toxicity of contrast agents [1-5]. Previous literature has reported several factors that contribute to the development of CIE. Hyperosmolar contrast agents are more likely to induce BBB disruption and cause CIE than lower or iso-osmolar contrast agents [2,3]. However, CIE also can be caused by lower or iso-osmolar contrast agents [4]. Other factors contributing to CIE include total amounts, low-temperature use of contrast agents, and short injection intervals [6,7]. In the past study, the mean dose of the contrast material in patients with CIE was approximately 250 ml [7]. Vasospasm and stagnation of contrast agents may also induce CIE [8,9]. Lesions of the posterior circulation may be at an increased risk because of the fragile BBB [10]. Hypertension and chronic kidney disease are known risk factors [1,4,11,12]. Chronic hypertension can impair cerebral autoregulation and may contribute to contrast leakage [4]. Chronic kidney disease may develop neurotoxicity of contrast materials as a result of delayed elimination of the contrast materials. In the present cases, both procedures avoided large amounts and low-temperature use of contrast agents, while both patients had hypertension, and frequent injections of contrast agents into the same vascular territory were conducted. 

Many patients with CIE present with focal symptoms immediately after procedures, but some patients have symptoms in the subacute phase. CT findings associated with CIE are local cortical enhancement, increased subarachnoid density, and brain edema [13]. Findings of MRI associated with CIE are hyperintensity areas on FLAIR, which differs from the observation of cerebral infarction [1]. In Case 1, T2-weighted images and FLAIR images revealed a high-intensity lesion in the right lateral medulla oblongata, while diffusion images of MRI demonstrated no ischemic lesions. The lesion was thought to have vasogenic edema and was diagnosed as CIE. In Case 2, post-operative CT revealed a high-density lesion in the left lateral medulla oblongata. The water-iodine image of dual-energy CT, however, demonstrated no lesion. The lesion was considered a contrast leakage and was diagnosed as CIE.

Treatment of CIE varies depending on the reports, and it remains controversial. CIE is often treated with hydration and pharmacotherapy suck as steroids, antiepileptic drugs, and control of intracranial pressure. Steroids are often used to improve cerebral edema and may stabilize the BBB. Most symptoms related to CIE disappear within 48-72 hours, and most patients with CIE have a good prognosis, except for a few reports of irreversible cases [14,15]. 

## Conclusions

We examined two cases of localized CIE of the medulla oblongata following endovascular treatment for lesions of the posterior circulation.

To prevent CIE, it is essential to avoid frequent injections, large amounts, low-temperature use, and stagnation of contrast agents during procedures. Patients with chronic kidney disease, hypertension, and lesions of the posterior circulation system are at risk of CIE, and particular caution should be required.

It is important to understand the mechanism and pathology of CIE for prompt diagnosis and appropriate treatment.
